# Massive uterine fibroid: a diagnostic dilemma: a case report and review of the literature

**DOI:** 10.1186/s13256-021-02959-3

**Published:** 2021-07-13

**Authors:** Wiesener Viva, Dhanawat Juhi, Andresen Kristin, Mathiak Micaela, Both Marcus, Alkatout Ibrahim, Bauerschlag Dirk

**Affiliations:** 1 Department of Gynecology and Obstetrics, University Medical Center UKSH, Campus Kiel, Arnold-Heller-Straße 3, Haus C, 24105 Kiel, Germany; 2 Spectrum Clinic and Endoscopic Research Institute, 6A and 6B Neelamber building, Shakespeare Sarani, Kolkata, West Bengal 700020 India; 3 Institute of Pathology, University Medical Center UKSH, Campus Kiel, Arnold-Heller-Straße 3, Haus C, 24105 Kiel, Germany; 4 Department of Radiology and Neuroradiology, University Medical Center UKSH, Campus Kiel, Arnold Heller Straße 3, Haus C, 24105 Kiel, Germany

**Keywords:** Uterine mass, Giant fibroid, Misdiagnosis, Surgery, Weight gain

## Abstract

**Background:**

Fibroids of the uterus are the most common benign pelvic tumors in women worldwide. Their diagnosis is usually not missed because of the widespread and well-established use of ultrasound in gynecological clinics. Hence, the development of an unusually large myoma is a rare event, particularly in first-world countries such as Germany. It is even more uncommon that a myoma is misdiagnosed as a dietary failure.

**Case presentation:**

Herein, we report the case of a Caucasian woman with a *giant* fibroid that reached a size of over 50 cm, growing slowly over the past 15 years, and was misdiagnosed as abdominal fat due to weight gain. We aim to discuss the factors that lead to the growth of such a huge tumoral mass, including misdiagnosis and treatment, and the psychological impact. Through this case, we intend to increase the awareness among general physicians and gynecologists. Although menstrual disorders incorporate several pathologies, adequate assessment remains the primary responsibility of health care providers. A literature review revealed approximately 60 cases of *giant* uterine fibroids.

**Conclusion:**

The use of clinical and diagnostic devices, especially ultrasound, in this case, is indispensable. In conclusion, the growth of a *giant* fibroid can have disastrous effects on a woman’s health, including surgical trauma and psychological issues.

## Introduction

Leiomyomas or fibroids are the most common benign pelvic tumors in females that grow monoclonally from the smooth muscle cells of the uterus. Such tumors occur in nearly half of women over the age of 35 years, with increased prevalence during the reproductive phase due to hormone-stimulated growth [1]. At 50 years of age, 80% of African and almost 70% of Caucasian women have fibroids [2]. As the underlying pathogenesis of the development of these tumors remains unclear, several risk factors, such as positive family history, genetic alterations, and lifestyle factors (smoking, obesity, dyslipidemia, nutrition, exercise, and medical contraception), have been identified. Treatment of these lifestyle-associated risk factors with vitamin D supplementation, statin use, and dietary modification appears to be protective, along with parity [1, 3]. Myomas may occur as a single lesion or as multiple lesions as reported in two-third of the cases, with variation in size from microscopic to large macroscopic extent [1, 4]. As the majority of women with myomas remain asymptomatic [2], the number of undiagnosed uterine fibroids is high. Symptomatic women most likely suffer from abnormal uterine bleeding (meno- or metrorrhagia and polymenorrhea) as well as dysmenorrhea. Other frequent symptoms include dyspareunia or chronic acyclic pelvic pain [3]. Fibroids affect fertility [5] and can have a severe psychological impact on a woman’s life [3]. With continued growth, myomas can cause compression-related symptoms, such as dyspnea, frequent urination, or bowel complaints. The growth rate of myomas varies intra- and interindividually, thereby regressing or gradually increasing in size until the climacteric period is possible [1]. The identification of rapidly progressing growing fibroids requires close observational ultrasound examinations. Extremely large myomas can involve serious complications such as respiratory failure due to diaphragmatic compression [6] or incarcerated abdominal wall hernia [7].

In Germany, universal access to healthcare services is guaranteed by law [8]. The German ambulatory care sector is densely structured with accessibility of general physicians in less than 30 minutes in more than 90% of all cases [9]. Utilization of gynecological services in Germany usually begins between the ages of 15 and 16 years [10] and continues at age 20 with annual visits for prevention of cervical carcinoma [11], followed by recurrent examinations for breast cancer prevention [12]. The self-reported prevalence of myomas is high in German women (8.0%), with a mean age of 33.5 years at diagnosis. After the USA, Germany has the second-highest hysterectomy rate among women with uterine fibroids (29.1% versus 21.8%) [3]. Although diagnosis of a *giant* myoma is difficult with several possible differential diagnoses, the majority of uterine myomas are confidently diagnosed in the (pre-)clinical routine [1]. Herein, we present a rare case of a German woman whose uterine tumor was misdiagnosed and remained untreated for the past 15 years, growing into a *giant* fibroid (16.4 kg) with a size over 50 cm.

## Case report

A nulligravid, 46-year-old German woman presented to the gynecology clinic because of abnormal uterine bleeding and a slowly increasing abdominal extent in the past 15 years. She had no bowel or bladder complaints. The patient reported two episodes of polymenorrhea and menorrhagia in the past years. Due to the patient’s general fear of physicians and absence of frequent symptoms, she consulted her gynecologist and general physician sporadically. The gynecologist did not use ultrasound to clarify the uterine pathology. The general physician attributed her progressive abdominal extent to weight gain and advised dietary change and physical exercise as management. Both primary health care providers did not perform a thorough physical examination, including imaging methods, leaving the fibroid undiagnosed and untreated.

**Fig. 1:**
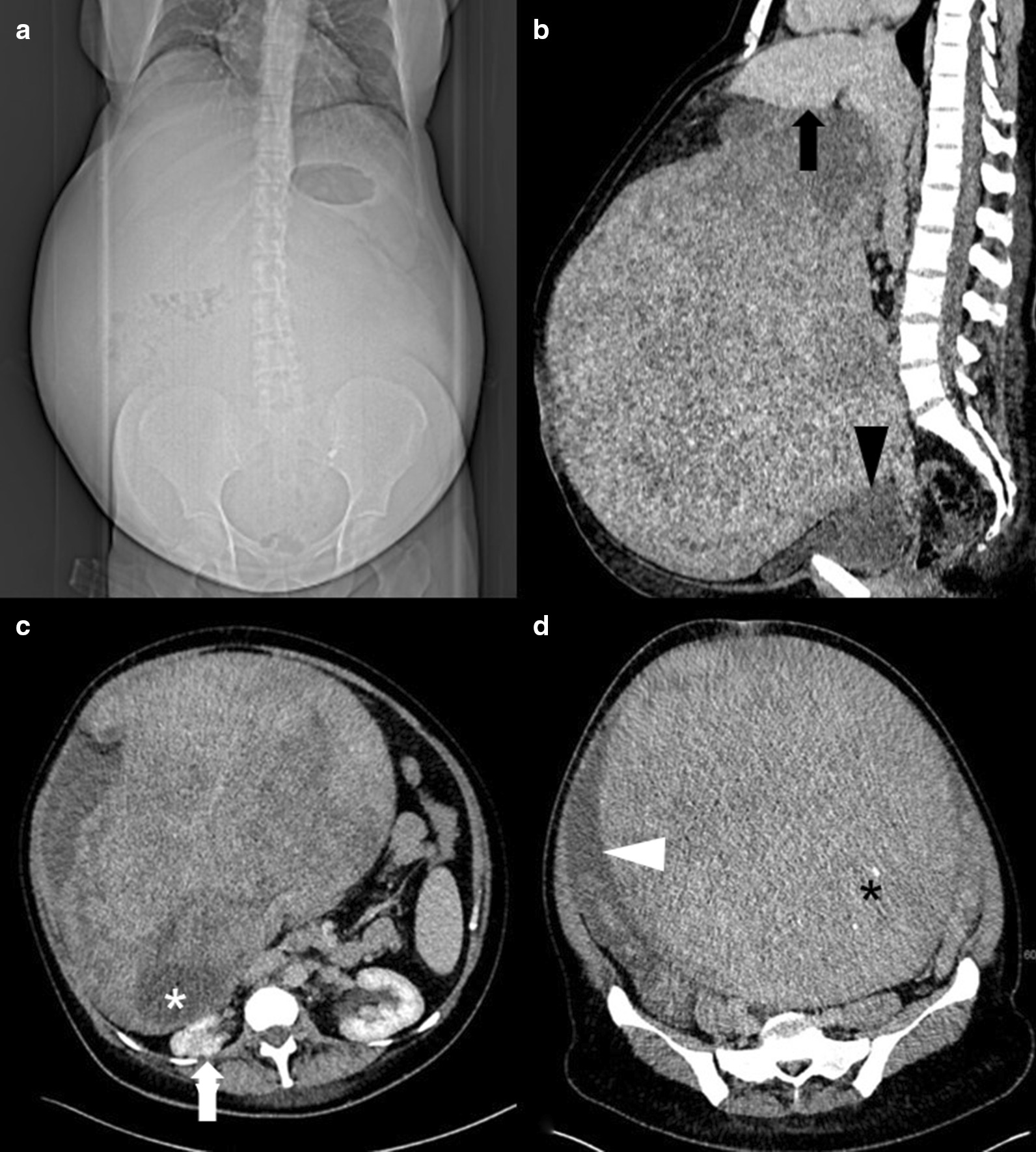
CT reveals extensive abdominal enlargement in the scout view (**a**). Sagittal CT reconstruction depicts a giant tumor in contact with the liver (black arrow, **b**) and with the urinary bladder (black arrowhead, **b**). The mass contains necrotic components (white asterisk, **c**), as well as small calcifications (black asterisk, **d**). The preoperative situs shows compression of the right kidney (white arrow, **c**) and ascites adjacent to the tumor (white arrowhead, **d**)

In our clinic, a preliminary physical examination was performed, which indicated good general condition and no evidence of pallor or pedal edema. The patient’s preoperative body mass index (BMI) was 32.1 kg/m^2^. Her abdomen was enormously enlarged and pendulous with flank fullness on both sides. An irregular mass arose from the pelvis up to the xiphisternum and was not discernible owing to abdominal wall obesity. There were no hernias or abdominal varices. Renal angle fullness was not observed. Because of the patient’s anxiety, a vaginal examination could not be performed. Transabdominal ultrasound showed a huge intraabdominal mass. The right kidney showed impaired cirrhosis, while the left kidney showed compensatory enlargement. A small amount of ascites was observed. An urgent computed tomography (CT) scan was performed revealing a large tumor that occupied the abdominopelvic cavity completely. On the CT scan, the mass measured 32 × 27 × 34 cm (intralesion diameter) and could not be visibly separated from the uterine cavity, bladder, or liver (Fig. 1). The tissue of origin and extent of tumor invasion remained unclear. The mass appeared heterogeneous, containing cystic and necrotic areas along with solid components. It compressed the intestines, right kidney, and both ureters. The spleen was mildly enlarged. The hepatorenal recess (Morison’s pouch) showed minimal ascites. No lymph nodes were observed. Due to the slow growth of the tumor, few ascites, and negative lymph nodes, malignancy was highly unlikely.

**Fig. 2:**
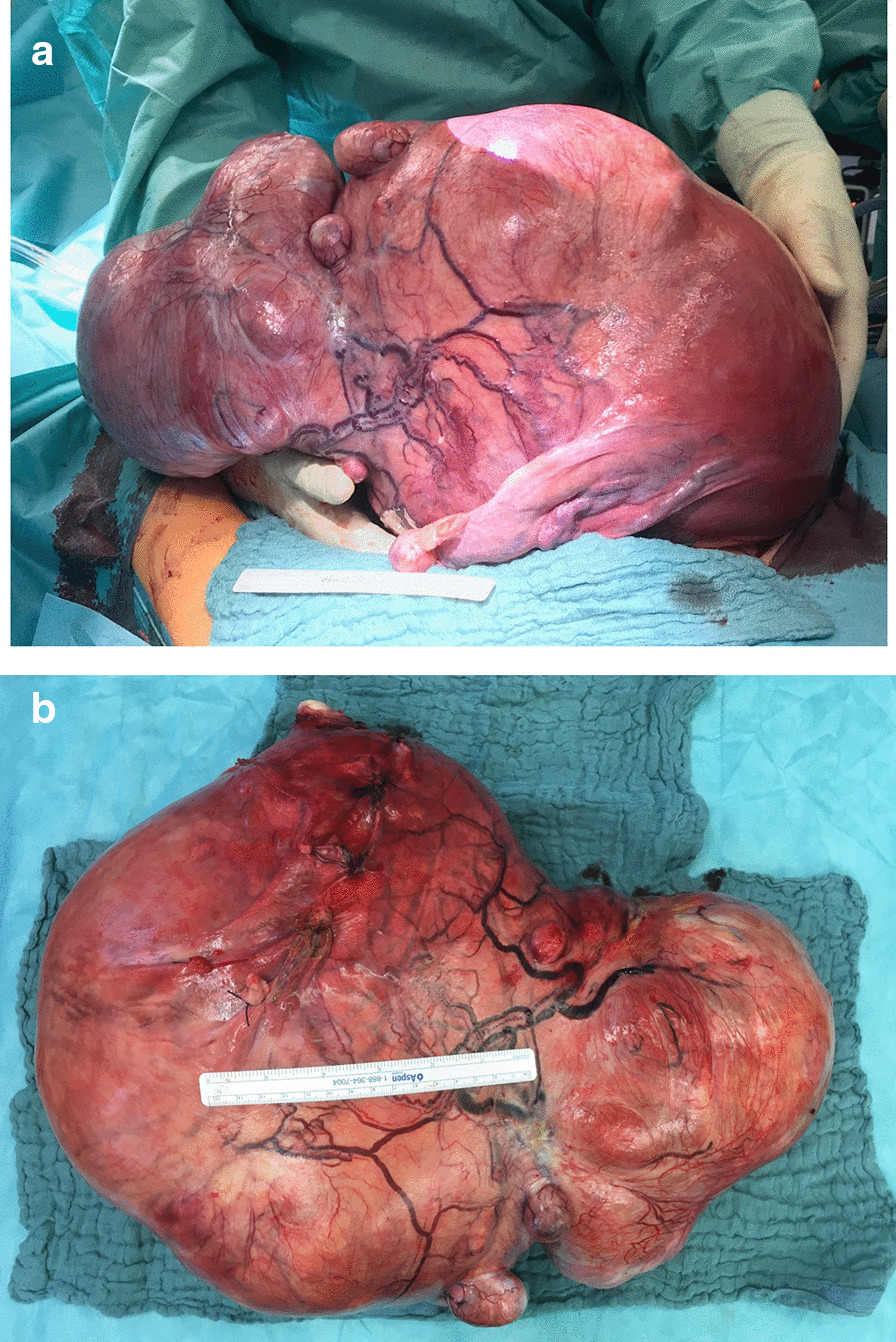
The tumor shows a dilated fallopian tube and an enlarged ovary (**a**). The fibroids appear macroscopically inhomogeneous with enlarged superficial vessels (**b**)

A midline longitudinal incision was made from the xiphisternum to the pubic symphysis, and the abdomen was opened. A large mass arising from the uterus up to the xiphisternum, firm in consistency with enlarged superficial veins, was seen. The mass extended laterally to both flanks and occupied the right and left hypochondrium. No adhesions to the intestinal organs were observed. The bilateral ovaries were enlarged to twice the normal size, with ovarian artery pulsation seen on both sides. Additionally, the bilateral fallopian tube round ligaments were thickened (Fig. 2a and b). Due to the *in situ* findings, a total abdominal hysterectomy *en bloc* with bilateral salpingectomy was performed, and both ovaries were left intraabdominally. Postoperatively, bilateral ureteric peristalsis was confirmed. Intraoperative blood loss was 400 ml. The patient’s postoperative clinical course within 5 days of hospital stay remained complication-free with quick recovery. She was discharged after 5 days of surgery and had good overall health.

Pathology confirmed a myomatous uterus measuring 52 × 37 ×  3 cm and weighing 16.4 kg. The tumor consisted of two separate myomas with diameters of more than 30 cm. Macroscopically, the shape was irregular, with overall consistency being firm with few soft areas. The tumor was pinkish-red in color, similar to (smooth) muscle cells. On the surface, enlarged aberrant blood vessels were observed. The cervix appeared normal, as well as bilateral fallopian tubes, although they were enlarged. For further histopathological examination, a cut section (total of 38 blocks) was performed, and tissue sections were stained with hematoxylin and eosin and examined under a light microscope. The cut sections revealed a heterogeneous phenotype with predominant white whirling structures. Microscopically, the tumoral mass consisted of smooth muscle cells and collagen bundles. Few areas had nuclear polyploidy, blood vessels, and enlarged glands with some superficial hemorrhagic areas. There was no evidence of malignancy.

## Discussion

Although uterine leiomyomas are frequent in women, fibroids > 50 cm in size, similar to the present case, with a weight of 11.6 kg (25 lb) and more being defined as *giant*, are exceedingly rare. The potential for benign tumors to outgrow quietly without causing specific symptoms is reasonable because of the large volume of the abdominal cavity, flexibility, and slow growth rate of the tumor [2]. The largest myoma ever reported weighed 63.3 kg and was discovered on autopsy [13]. Online search using the *PubMed* database showed approximately 60 cases of *giant* uterine myomas in the past 50 years worldwide [14]. Table 1 summarizes the global cases of *giant* uterine fibroids in the past 20 years.

**Table 1:** Overview of cases with *giant* uterine myomas in the last 20 years

Year	Total tumor weight (kg)	Tumor size (cm)	Age (years)	Key symptoms	Reference country
*2020*	*16.4*	*52.0/37.0/33.0*	*46*	*Abnormal uterine bleeding, abdominal distension*	*Present case* *Germany*
2018	27.8	64.0/50.5/15.0	53	Dyspnea	[20] Singapore
2016	11.6	43.0/32.0/23.0	46	Abdominal distension, lower abdominal pain, urinary frequency, dyspnea, peripheral edema	[23] India
2016	15.6	Not available	42	Not available	[20] Czechia
2015	20.0	33.0/28.0/22.0	40	Back pain, abdominal pressure and distension, weight loss, constipation, urinary frequency	[25] India
2014	28.1	62.0/39.0/21.0	39	Not available	[18] Greece/ Germany
2014	16.8	(1) 24.0/32.0 (2) 4.5/6.3 (3) 6.0/6.2 (4) 5.4/8.7	31	Infertility, weight loss, dyspnea on exertion	[26] Nigeria
2011	11.6	31.0/26.0/14.0	33	Anemia, abdominal distension	[2] Texas
2011	18.1	33.0/28.0/22.0	45	Back pain, abdominal pressure and distension, weight loss, constipation, urinary frequency	[27] Rumania
2008	27.7 (1)15.3 (2) 12.4	(1) 26.0/20.0/18.0 (2) 20.0/17.0/16.0	55	Abdominal distension, tiredness, difficulties with physical movement	[28] Italy
2005	12.4	Not available	45	Constipation, dyspnea	[29] Mexico
2003	13.0 (27 kg of ascites)	52.0/33.0/22.0	54	Difficulties with physical movement	[6] Israel
2003	43.0	61.0/53.0/26.0	49	Dyspnea, respiratory failure	[6] Israel

Preoperative imaging studies are useful to define the extent of the tumor and to assess the likelihood of malignancy in cases of expansive or infiltrative growth. Ultrasonography is the preferred technique for the initial evaluation of gynecologic pathology because of its ubiquitous availability, noninvasiveness, and convenient cost–benefit ratio [15]. In the present case, preclinical ultrasound imaging would have been absolutely appropriate with regard to diagnosis, surveillance, and prevention of myoma-associated complications. As fibroids continue to grow, they outgrow their blood supply. Therefore, *giant* myomas often undergo degenerative changes, and dystrophic calcification can complicate the diagnosis [16]. Although a CT scan may not be the preferred method, many myomas are detected incidentally by CT imaging [15]. The widespread clinical use of a CT scan lies in its availability, time saving, and comfortable use. Lastly, magnetic resonance imaging (MRI) is recommended to define and measure uterine pathology confidently. As our patient was claustrophobic, MRI was not suitable for her. This imaging method is predominantly utilized in first-world countries in maximum-care hospitals because of its high cost. The atypical appearance of fibroids substantially limits the preoperative informative value of all techniques [15, 16]. Hence, the underestimation of the presented fibroid was due to its histologic composition that did not allow precise separation from the intestinal organs.

Uterine leiomyomas have been misdiagnosed as adenomyosis, hematometra, uterine sarcoma, ovarian masses, and pregnancy [15, 17, 18]. Other common non-gynecological differential diagnoses include gastrointestinal tumors or inflammation [19]. Fibroids often occur with endometriosis and adenomyosis, with an overlap of symptoms [20], which significantly reduces diagnostic confidence. The position of the fibroid in relation to the uterus affects the patient’s symptoms and diagnostic specificity. Myomas occur within the muscular layer (70% of all cases; intramural), on the outside (20% of all cases; subserosal), or the inside (10% of all cases; submucosal) of the uterine cavity where they possibly have a connective stalk (pedunculation). Pedunculated subserosal myomas can be acutely symptomatic owing to torsion with obstruction of blood vessels, which requires immediate surgery. They often mimic the ovarian pathology. Another differential diagnosis is uterine cancer, with carcinomas being the most frequent and sarcomas and carcinosarcomas occurring rarely [2]. Malignant transformation of a leiomyoma to a leiomyosarcoma occurs in 0.2% of all cases [16]. It should be stressed that no imaging method can rule out malignancy so far, leaving the diagnosis of a *giant* uterine fibroid a challenge. Fibroids of an enormous extent cannot be treated with the most widely used minimally invasive surgery techniques: hysteroscopic myomectomy, vaginal hysterectomy, or total laparoscopic hysterectomy (TLH)/laparoscopic-assisted supracervical hysterectomy (LASH). Similar to the present case, the majority of *giant* fibroids are removed during total abdominal hysterectomy with additional bilateral salpingo-oophorectomy, depending on the patient’s age and affection of both adnexa. Intraoperatively, severe complications such as hemodynamic instability can occur because of extensive blood loss [2, 21]. With regard to the amount of surgery, the general morbidity and mortality in patients who receive a laparotomy is remarkably higher. Postoperative complications include venous thrombosis and acute renal failure [22]. Generally, *giant* myomas are fatal for the patient; therefore, such patients have to be treated similarly to older multimorbid patients [2], with death being a possible outcome [23].

The prevention of *giant* fibroid development with close surveillance and early surgical therapy for women with progressive myomas is the clinical gold standard. In Germany, uterine fibroids indicate surgical hysterectomy in 60.7% of all cases [20]. This underlies the fact that uterine tumors are a relevant reason for hospitalization in women. The development of such a *giant* myoma in the present case is surprising despite the easy accessibility to professional care and high educational standard of the population in Germany. According to Stentzel *et al.*, the utilization of professional care depends on several personal factors rather than travel time. In particular, a high socioeconomic status was positively correlated with visits to gynecological care [9]. Data from the cross-sectional German Health Survey (GEDA) indicate that low social status correlates with less participation in medical check-ups [24]. This strengthens the role of education in the requirement of self-consciousness and awareness of health checks.

Given the patient’s unemployment for the last 3 years and her modest family background, her low socioeconomic status could have contributed to her worsening condition. Additionally, her general anxiety and previously diagnosed depressive state of mind could have led to the rejection of professional care. The misdiagnosis by her previous doctors could be explained by her lack of complaint regarding irregular menstruation. Women with fibroids of this size are expected to most likely suffer from menstrual disorders [1], but the patient presented with menstrual irregularities only twice in the past 15 years. This possibly did not prompt her attending physicians to further evaluate the uterus as a cause of the irregular increase in abdominal size. This case was challenging to us as fibroids of this enormous size are rare, and hence, the first diagnosis of fibroid uterus was not made. Instead, it was suspected to be an ovarian carcinoma. Surgical challenges of access, intraoperative determination of anatomy, and hemorrhage were anticipated. Such large masses with uncertain diagnoses pose challenges for young and experienced surgeons alike. The patient was relieved after her treatment and was extremely thankful that she was acknowledged and not merely told that her problems were due to weight gain.

## Conclusion

Preclinical utilization of the services of gynecologists in northern Germany depends on personal factors, such as family background, educational level, and socioeconomic status. Menstrual disorders are diverse in diagnosis and have organic and nonorganic reasons that require diagnostic clarification. Therefore, liberal utilization of physical and ultrasound examinations by general physicians could help prevent a delay in diagnosis and therapy of treatable causes such as fibroids. *Giant* fibroids remain a diagnostic and surgical challenge, requiring expertise and interdisciplinary cooperation. Nevertheless, these gigantic benign tumors can be managed complication-free with proper diagnosis and surgical expertise.

## Data Availability

Data sharing is not applicable to this article as no data were collected or analyzed>.

## Abbreviations

BMI: Body mass index
CT: Computed tomography
MRI: Magnetic resonance imaging
TLH: Total laparoscopic hysterectomy
LASH: Laparoscopic-Assisted supracervical hysterectomy
GEDA: Gesundheit in Deutschland Aktuell
